# Improving Psychiatric Care for Adults with Intellectual and Developmental Disabilities (IDD)

**DOI:** 10.1192/j.eurpsy.2025.1554

**Published:** 2025-08-26

**Authors:** H. M. Nguyen, S. Joseph, M. Liu, B. Carr

**Affiliations:** 1 University of Florida College of Medicine, Gainesville, FL, United States; 2 Psychiatry, University of Florida College of Medicine, Gainesville, FL, United States

## Abstract

**Introduction:**

Adults with intellectual and developmental disabilities (IDD) often struggle to access suitable psychiatric care due to cognitive and communication challenges. Traditional mental health services are not always well-equipped for these needs, highlighting a critical need for evidence-based, IDD-specific guidelines. Expanding research, especially randomized controlled trials (RCTs), is essential to develop personalized, effective treatments for this population.

**Objectives:**

This poster aims to outline current gaps in psychiatric care for adults with IDD, propose strategies for treatment adaptation, and emphasize research priorities to advance evidence-based care for this underserved group.

**Methods:**

A literature review assessed studies on treatment adaptations for IDD, focusing on the effectiveness of current interventions and limitations in existing research. Priority was given to innovative care approaches and adaptations in standard mental health treatments.

**Results:**

Psychiatric care for adults with IDD often involves adapting treatments like cognitive-behavioral therapy (CBT) and pharmacotherapy with simplified, patient-centered methods. For example, CBT adaptations may include breaking techniques into smaller steps, using visual aids, and shorter sessions, which accommodate cognitive limitations. Personalized care is essential due to varying abilities within this population. Pharmacological treatments also require careful titration and close monitoring, as the risk of side effects is higher among those with IDD. However, few RCTs assess the efficacy and safety of treatments for severe psychiatric disorders in the IDD population, underscoring the need for more rigorous research to support clinical guidelines. To overcome recruitment and follow-up barriers in RCTs, studies suggest alternative methods such as targeted outreach through day centers, provider organizations, and one-on-one approaches, which have been effective in reducing dropout rates. Furthermore, bipolar disorder treatment in IDD is particularly under-researched, despite its significant impact on quality of life, highlighting a need for focused studies in this area.

**Conclusions:**

Enhancing psychiatric care for adults with IDD will require systemic changes, including increased research funding, IDD-specific clinical guidelines, and improved provider training. RCTs focused on adapted therapies and pharmacological interventions are crucial to build a strong evidence base. By promoting individualized, evidence-based approaches, the field can better meet the unique needs of adults with IDD, ultimately improving mental health outcomes and quality of life.

**Disclosure of Interest:**

None Declared

